# Onset of Sensory Function and Embryonic Discrimination of Ecologically Relevant Cues in Fish

**DOI:** 10.1093/iob/obag036

**Published:** 2026-07-08

**Authors:** K Steinberg, J Ward

**Affiliations:** Department of Biology, Ball State University, Foundational Sciences Building, 1600 Ashland Ave, Muncie, IN 47306, USA; Department of Biology, Ball State University, Foundational Sciences Building, 1600 Ashland Ave, Muncie, IN 47306, USA

## Abstract

Embryos of oviparous species use a variety of sensory modalities to perceive and respond to environmental cues. However, the timing and mechanisms by which prenatal sensory experiences are acquired, encoded, and translated into lasting behavioral outcomes remain poorly understood. Here, we identified the developmental window of the onset of behavioral responses to olfactory cues in a teleost model of embryonic behavioral development, the fathead minnow (*Pimephales promelas*), and tested the hypothesis that embryos are sensitive to differences in the information content of cues indicative of predation on eggs vs. free-swimming conspecifics. Assays conducted at regular intervals during embryogenesis showed that a behavioral response to olfactory cues emerges well before hatching, with individuals responding to cues that indicate egg threat. Embryonic exposure to olfactory cues indicative of predation did not significantly alter the timing of emergence from the egg or larval behavior after hatching, but individuals reared in the presence of olfactory cues from injured conspecifics tended to hatch at an earlier stage of development. Our findings suggest that the timing of cue exposure relative to sensory development during embryogenesis may be an important factor contributing to behavioral plasticity in oviparous animals.

## Introduction

Fishes and other aquatic organisms rely on information acquired through multiple sensory modalities to interpret and respond to environmental challenges and opportunities (Maaswinkel and Li 2003; Stynoski and Noble 2012; Coombs et al. 2014). However, how an individual perceives, processes, and integrates sensory cues is shaped by a combination of prior and current factors, including the organism’s physiology and development, current environmental conditions, and the evolutionary history of the species (New and Kang 2000; Collin 2007; Preininger et al. 2013). For example, cave-dwelling fishes such as the Mexican characid (*Astyanax mexicanus*) rely more heavily on mechanosensory input from the lateral line system to navigate the aphotic subterranean habitat than related species that live at the surface (Soares and Niemiller 2013). Cave-dwellers also have larger olfactory organs (Soares and Niemiller 2013) and are more sensitive to odors than surface-dwelling *Astyanax* species (Hinaux et al. 2016). Within species, the relative dominance or sensitivity of different sensory modalities can vary seasonally, or with sex, age, or experience. For example, female plainfin midshipman (*Porichthys notatus*) show increased auditory sensitivity during the breeding season that improves detection and localization of male vocalizations (Sisneros 2009). These examples emphasize how the sensory ecology landscape shapes behavioral plasticity in a variety of fitness contexts (Lickliter 2005; Jordan and Ryan 2015).

Growing evidence also indicates that aquatic organisms detect and process environmental stimuli before hatching, enabling them to respond to immediate changes in the local environment, and/or to acquire information relevant to later stages of ontogeny (Kusch and Chivers 2004; Romagny et al. 2012; Kempster et al. 2013; Kokel et al. 2013; Mezrai et al. 2020; Crowder and Ward 2022). For example, embryonic cuttlefish (*Sepia officinalis*) visually exposed to specific prey items exhibit preferences for those same prey types after hatching (Darmaillacq et al. 2008). Several fish and frog species are capable of detecting and recognizing olfactory predator cues as embryos, and may exhibit behavioral responses to those cues before and/or after emergence (Mathis et al. 2008; Nelson et al. 2013; Atherton and McCormick 2015; Gazzola et al. 2023; Karasch and Ward 2025). Yet, despite the fact that the embryonic sensory environment is known to influence the development of behavior, how these early sensory experiences are acquired, encoded, and translated into lasting behavioral outcomes remains poorly understood (Caldwell et al. 2010; Jung et al. 2022).

One area of limited understanding is how the onset of sensory system function might constrain behavioral development and the establishment of longer-term patterns of behavioral organization. An embryo’s ability to respond to an environmental cue depends on sufficient development of the sensory system required to detect the stimulus, the neural architecture necessary to process the information, and the musculoskeletal system needed to execute that response. Thus, the extent to which prenatal sensory experience shapes behavioral development may depend on the timing of exposure relative to the embryo’s sensitivity to the type, and/or intensity and relevance of the stimulus (Easter, Jr. and Nicola 1996; Majoris et al. 2021). The sensitivity with which embryos can discriminate among cues within the same ecological context that differ in informational content is also little known, although natural selection should favor the ability of individuals to modulate behavioral responses in ways that are appropriate to the context (Caldwell et al. 2010; Ferrari et al. 2010b; Warkentin 2011b). Consistent with this hypothesis, embryos of some oviparous aquatic species do show evidence of fairly sophisticated cue discrimination (Warkentin 2005; Ferrari et al. 2010b; Garcia et al. 2017). For example, embryonic red-eyed treefrogs (*Agalychnis callidryas*) demonstrate escape-hatching in response to vibratory frequencies typical of those induced by a snake during an attack on the clutch, but not to vibratory frequencies consistent with rainfall (Warkentin 2005). Indeed, embryos should be particularly discriminatory with respect to cues indicative of predation because the optimal behavioral response may differ depending on the immediacy and severity of the threat. For example, whereas “freezing” may be an appropriate response to a perceived predator prior to detection (Kempster et al. 2013), “escape” may be the only behavioral option available to prey once detection has occurred (Eilam 2005).

Related evidence also indicates that prenatal or pre-hatch exposure to predator cues may also induce plasticity in morphology, development, and hatching processes (West-Eberhard 2003; Mandrillon and Saglio 2009; Nettle and Bateson 2015; Meuthen et al. 2019b; Gazzola et al. 2023). Embryos exposed to predator-related cues may modulate the timing of hatching according to the information content of those cues, delaying emergence when external predation risk is high and hatching prematurely when remaining in the egg poses a greater risk (Warkentin 2011a, 2011b; Jung et al. 2020). However, further research is needed to understand how sensory cue processing during embryogenesis contributes to developmental plasticity in morphology, hatching, and behavior both before and after egg emergence.

The goal of this study was to improve our understanding of how the pre-hatch sensory environment shapes early perceptual, behavioral, and cognitive development in oviparous aquatic vertebrates by determining when embryos of a representative species first express behavioral responses to environmental information, if they show evidence of cue discrimination, and whether the contextual relevance of available environmental cues influences developmental plasticity and longer-term (post-hatch) patterns of behavior. To achieve this goal, we conducted a time-series experiment (Experiment 1) using a common freshwater fish, the fathead minnow (*Pimephales promelas*) to determine the developmental window(s) during which embryos first express behavioral responses to external olfactory stimuli. Fathead minnows are an excellent species for testing general questions related to the development of behavior because embryonic development in *P. pimephales* is relatively well-characterized, with individuals passing through 32 distinct developmental stages before hatching (Devlin et al. 1996; Böhler 2021). We presented embryos with chemical alarm cues generated from injured adult conspecifics or 5-day-old eggs at 48- [stage 25–26], 72- [stage 27–28], 96- [stage 30–31], and 120-h post-fertilization (hpf) [stage 32] and observed behavioral responses to the stimuli at each timepoint. In a separate experiment (Experiment 2), we manipulated the pre-hatch olfactory environment to evaluate potential differences in hatching, development, and post-hatching behavior associated with differences in perceived predation risk (i.e., predation upon eggs vs. free-swimming adult conspecifics). To do this, we exposed fathead minnow clutches twice a day to chemical alarm cues from adults or eggs, and compared embryos reared under perceived predation risk to eggs or hatched fish with those reared in the absence of these cues.

## Materials and methods

### Animals, breeding, and maintenance

Predator-naïve, sexually mature fathead minnows were acquired from a culturing facility (Aquatic Biosystems, Inc., CO, USA) and housed in a 530-L living stream (Frigid Units, Inc., OH, USA) divided into three compartments by opaque mesh barriers. Males and females were divided by sex and kept in the distal compartments of the stream, with an empty compartment between them. All animals were maintained under summer breeding conditions consisting of a 16-h light to 8-h dark photoperiod, and water temperature varied from 19 to 23°C. The fish were fed bloodworms and brine shrimp (*Artemia* spp) twice a day.

Male and female fathead minnows were randomly selected from the living stream and allocated to 6-L breeding tanks in a recirculating housing unit (Aquaneering, Inc., CA, USA) to generate eggs. Each tank contained a pair of fish consisting of one male and one female that was visually and physically separated from other pairs. The tanks were each equipped with a spawning tile made from a half section of polyvinyl chloride (PVC) lined with a removeable piece of flexible polyethylene sheeting. The tanks were checked twice a day for eggs, and spawning tiles with eggs were removed immediately upon discovery and placed in 500-mL glass vessels filled with fresh water and containing an air stone. A total of 46 breeding pairs generated eggs for this study (11 for Experiment 1 and 35 for Experiment 2). Each pair of breeding fish was only used once to generate one clutch. Breeding pairs that did not generate eggs within a week were replaced. For Experiment 1, cameras (GoPro Hero 12, San Mateo, CA, USA) positioned underneath the breeding tanks took a photo of the spawning tile every 5 minutes and the photos were reviewed to identify the precise time of spawning (within a 5-min window). All animal husbandry and protocols were performed in accordance with Ball State University’s Institutional Animal Care and Use Committee (Approval # 1142896-1).

### Preparation of olfactory cues used in Experiments 1 and 2

Alarm cue from adult minnows was generated from the epithelial tissue of 15 sexually mature female fathead minnows using the production method described in Minichiello et al. (2026), which was modified from previously published procedures (Mathis and Smith 1993; Cashner 2004; Crowder and Ward 2022; Karasch and Ward 2025). Males were not used because previous research has suggested that fish with high testosterone levels (characteristic of breeding males) secrete reduced alarm cue (Smith 1973). To create the cue, the fish were euthanized with MS-222 and rinsed thoroughly with cold water. Previous work in this species has indicated that MS-222 does not affect cue perception (Crowder and Ward 2022; Karasch and Ward 2025; Minichiello et al. 2026). A scalpel was used to remove the head, tail, and internal tissues, and the torsos were measured from standard length (mean ± SD: 3.49 ± 0.32 cm) and weighed (mean ± SD: 0.74 ± 0.18 g) to ensure that the individuals used to generate the preparation were of similar size. The skins were homogenized in a blender with an initial 50 mL of deionized water (DI), followed by the addition of another 350 mL of DI water to generate a stock solution of alarm cue with an approximate concentration of 0.03 g of tissue per mL, which represents 0.04 fish-equivalents per mL (Minichiello et al. 2026). The solution was filtered twice using fine mesh to remove tissue fragments, and was then pipetted into 1-mL aliquots and frozen at −20°C.

A 0.05 g of tissue per mL stock solution of embryonic alarm cue was created from fertilized, injured eggs aged 5 dpf according to the following formula: 1 g of eggs (corresponding to approximately 688 individual eggs) per 20 mL of water. To create the cue, eggs were weighed and homogenized using a mortar and pestle with 1 mL of DI water. Additional water was then added reach the appropriate calculated final volume. The solution was homogenized again, strained through fine mesh, pipetted into 1-mL aliquots, and frozen at −20°C.

### Experiment 1: Ontogeny of behavioral responses to external olfactory stimuli

After a clutch was removed from a breeding tank, it was divided into subgroups containing 5–25 fertilized eggs using a split-clutch experimental design. Each subgroup was assigned to a treatment and placed in a separate rearing vessel containing fresh water and an air stone. To divide the clutch, the spawning substrate was cut into approximately 1 × 1 cm sections with sterilized scissors within 20 h of spawning, with spawning time precisely determined from timelapse footage. Following sectioning, spawning tile fragments were rinsed with clean water to remove any substances released from damaged eggs before being transferred to the rearing vessels. However, it is unlikely that cues released during the cutting process influenced embryo development, because the sensory structures required for the detection of olfactory and mechanosensory stimuli (e.g., olfactory grooves, olfactory pits, and neuromasts) are not yet developed at this stage (Devlin et al. 1996). Starting at 48 hpf, and at 24-h intervals thereafter (i.e., 48, 72, 96, and 120 hpf) embryos were tested for evidence of a behavioral response to chemical alarm cues from conspecific eggs or adults. We did not test animals at 24 hpf because the olfactory and muscular systems are little developed, and pilot testing showed no evidence of embryonic movement at 24 hpf. To begin a test, a spawning sheet containing embryos was suspended 2.5 cm from the bottom of an 8.5-cm diameter plastic arena with an alligator clip. The arena contained 90 mL of aged, aerated water. The arena was placed under a stereomicroscope (Stemi 508, *Zeiss*, Jena, Germany) equipped with a microscope camera (Axiocam 208 color, *Zeiss*, Jena, Germany) and the embryos were permitted to acclimate for 3 min before testing, which is sufficient for embryos to return to normal activity after being moved (Crowder and Ward 2022; Karasch and Ward 2025).

Evidence for embryonic stimulus perception and response was evaluated by comparing the activity levels of embryos before and after exposure to a stimulus. We quantified activity as the number of “bursts” of spontaneous locomotor movement (e.g., flexing or rolling) made by an embryo within the egg (Crowder and Ward 2022; Karasch and Ward 2025). At the start of a test, the embryos were videorecorded for 1 min to measure baseline levels of embryonic activity. Embryos were recorded for an additional 1 minute after administration of the stimulus. The embryos were returned to their home vessel after each test. For most trials, 10 embryos from each fragment of spawning sheet were randomly selected for behavioral analysis, however small clutch sizes reduced the number of individuals available for observation in a few cases. Embryonic activity was extracted from recordings using an automated behavioral tracking system (DanioScope, Noldus Information Technology, Wageningen, The Netherlands).

Olfactory stimuli were administered following protocols from Crowder and Ward (2022), adapted for pre-hatched fish. Airline tubing fixed to the bottom of the dish and connected to a 12-mL syringe outside of the testing arena was used to deliver the olfactory stimulus. To administer the cue, 3 mL of the assigned treatment (adult alarm cue or injured egg cue) was gently injected into the tubing using a syringe. An additional 5 mL of water was subsequently gently injected into the tube to flush the cue into the arena. Each treatment had a separate dedicated arena and stimulus delivery system. To prevent mechanical disturbance of the embryos, cues were introduced to the arena below the polyethylene spawning substrate, and 20 s were allowed to disperse and penetrate the chorion. Embryonic activity was then recorded for 1 minute. Preliminary tests with clean water were conducted during the development of the assay to confirm that delivery of the water stimulus itself did not induce a behavioral response. Analysis confirmed that there was no effect of stimulus administration at any timepoint (Generalized Linear Mixed Model [GZLMM]: 48 h; 72 h; 96 h; 120 h: all *P*s between 0.77 and 1.0).

### Statistics

All analyses were performed using R Statistical Software (v4.3.3; R Core Team 2024). Preliminary screening indicated that embryonic activity data violated some parametric assumptions. Therefore, to determine the stage of development at which embryos first show evidence of a behavioral response we compared the activity levels of individual embryos before and after the administration of each stimulus at four developmental time points using GZLMMs [*glmmTMB* and *car* packages in R (Brooks et al. 2017; Fox and Weisberg 2019)] with a negative binomial distribution and a log-link function. This approach accounts for zero-inflation and overdispersion commonly observed in count data (O’Hara and Kotze 2010). The number of discrete bursts of locomotor activity performed by embryos was specified as the dependent variable. Developmental time point (48, 72, 96, or 120 hpf) and stimulus administration (pre-administration or post-administration) were included as fixed effects, along with their interaction. In addition, because embryos from each clutch were distributed among experimental tests, clutch identity was included as a random effect to account for the non-independence of observations arising from shared parentage (Bolker et al. 2009). The significance of fixed effects was assessed using Type III Wald statistics.

### Experiment 2: Influence of embryonic olfactory environment on hatching, development, and post-hatch behavior

Clutches were randomly assigned to one of three embryonic rearing treatments (i.e., exposure to adult alarm cue, injured egg cue, or a control treatment consisting of aged, clean water). The breeding tile containing eggs was gently removed from its housing container twice daily and placed in a 300-mL glass vessel containing 250 mL of aged, aerated water amended with 1 mL of the assigned cue (adult or egg) or clean, fresh water (control eggs) for 30 min each session. Clutches underwent these exposures for 5 consecutive days, beginning on the day they were laid. At the conclusion of an exposure session, the breeding tile was gently returned to its home container.

### Developmental and hatching plasticity

**Fig. 1:**
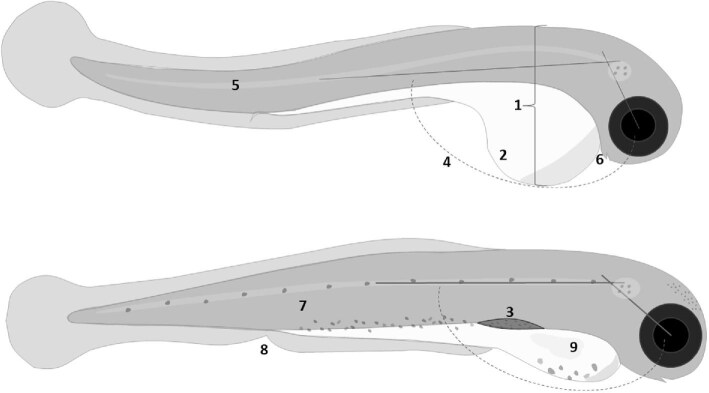
Location and assessment of morphological traits used to evaluate the development of hatchlings. The top figure visually represents a individual at an earlier stage of development and the bottom figure represents an individual at a later stage of development. (1) Dorsoventral diameter of the yolk; (2) shape of the yolk sac; (3) presence of swim bladder; (4) orientation of the head (lateral midline from center of eye to where the notochord meets the otic capsule) relative to the notochord (from midbody to where the notochord meets the otic capsule); (5) longitudinal body axis; (6) presence of dentary lower jaw differentiation; (7) presence of dorsal trunk melanocytes; (8) presence of an indentation in median fin fold at the cloacal opening; and (9) presence of yellow bile.

The eggs were checked for evidence of hatching twice daily to determine whether embryos exposed to cues differing in information content varied in the rate of development. The date and time of the first hatched egg was recorded as the minimum duration of development before evidence of spontaneous hatching. To assess whether early-emerging individuals from clutches reared under different olfactory conditions differ in phenotype, morphological measurements were collected from the first one to three larvae to emerge from each clutch. These larvae were euthanized via an overdose of MS-222, and photographed in lateral view under a stereomicroscope (Stemi 508, *Zeiss*) equipped with a microscope camera (Axiocam 208 color, *Zeiss*) in the presence of a 1-mm stage micrometer with a resolution of 0.01 mm. The larvae were measured for total length (TL) and compared by eye to a published developmental series for fathead minnows to determine the stage of development at the time of hatching (stages 28–32; with spontaneous hatching typically occurring at stages 31–32 at room temperature; Devlin et al. 1996). In addition, we classified each fish at hatching as fully developed, underdeveloped, or severely underdeveloped by quantifying the development of nine morphological traits that change in a predicable direction over this range of development using discrete binary categorizations. Each trait for each fish was independently scored as either “0” or “1,” with “0” representing the form of the trait earlier in development (Devlin et al. 1996). We used these nine traits and their binary classifications: (1) dorsoventral diameter of the yolk sac [greater than (0) vs. equal to or less than (1) the width of the body]; (2) shape of the yolk sac [round (0) vs. elongated (1)]; (3) swim bladder [absent (0) vs. present (1)]; (4) orientation of the head in comparison to the notochord is [smaller than (0) vs. larger than or equal to (1) a 140° angle at the point of intersection]; (5) longitudinal body axis [curved (0) vs. straight(1)]; (6) dentary lower jaw [not separated (0) vs. separated from body (1)]; (7) dorsal trunk melanocytes [absent (0) vs. present (1)]; (8) indentation in median fin fold at the cloacal opening [absent (0) vs. present (1)]; and (9) yellow bile [absent (0) vs. present (1)] (Fig. 1). The trait scores for each fish were subsequently summed. Individuals that scored 7 or more were classified as “fully developed” (corresponding to stages 31–32); those that scored 3–6 were classified as “underdeveloped” (stage 30), and those that scored 0–2 were classified as “severely underdeveloped” (stages 28–29).

Hatching was monitored until approximately 90% of the eggs had hatched, and the date and time were recorded to calculate the duration of the hatching period (days elapsed from first to last hatch). The larvae were reared in 500-mL glass vessels containing aged, aerated water and an airstone until 21 dpf, at which time they were tested for evidence of behavioral carryover effects in two behavioral assays. The larvae were fed brine shrimp twice a day and monitored for mortality daily.

### Open-field trials

Larvae were tested in open-field trials at 21 dpf, which exploit fishes’ innate preference for thigmotaxis (wall-hugging) and are an established method for measuring organismal boldness (Walsh and Cummins 1976; Maximino et al. 2010; Dahlbom et al. 2011). To begin a test, five larvae were randomly selected from each clutch and placed in separate circular Plexiglas arenas (4-cm diameter) containing 15 mL of clean, aerated water. The walls of each arena were covered with white opaque tape and the bottom was transparent. A 2-mm grid on the bottom of each arena allowed for calibration. The five arenas were placed on a dimmable LED light board centered below a GigE camera (Basler AG, Germany). After a 3-min acclimation period, the trial was started and the behavior of larvae in the arena was recorded for 2 min.

The swimming behavior of the fish was analyzed using Ethovision XT behavioral tracking software (Noldus XT, Wageningen, The Netherlands). Before analysis, the arena was divided into a central zone with a diameter of 1.5 cm, and an outer zone including the perimeter of the arena. For each fish, the total distance traveled during the trial (mm) and the mean swimming velocity (mm/ms) were automatically extracted from the tapes. We also recorded the amount of time (s) that each fish spent in the central and outer zones, respectively, and the number of times it entered the central zone. In this assay, higher values of swimming activity (measured by the distance swam) and more time spent in the center of the arena were used as evidence of greater boldness in fish (Sneddon 2003; Maximino et al. 2010).

### Refugium trials

Differences in larval risk-taking behavior associated with developmental rearing environment were also tested in refugium emergence trials (Garcia et al. 2017; Meuthen et al. 2019a), modified for larval fish. Five circular arenas (5-cm diameter) were used for this experiment. Approximately one-third of the arena consisted of an isolation chamber (refugium) consisting of opaque white sides and top. The walls of the remaining two-thirds of the arena were transparent and the top of the arena was open to the environment, creating the perception of an expansive space. Passage between the refugium and open areas of the arena was facilitated by an opaque plastic divider that could be manually removed. The arenas were positioned on top of a dimmable LED light pad such that the opaque portions of the arena walls faced each other and there was no visual contact between individuals in different arenas during trials. At the start of a trial, a fish was placed in the refugium of each arena and permitted 5 min to acclimate to the space. The plastic divider was then removed, and the arenas were recorded with a GigE camera (Basler AG, Germany) for 10 min. The time (in seconds) for each fish to emerge from the protected area was manually extracted from the tapes by one researcher, with emergence time recorded when at least half of the fish was visible (i.e, the anterior-posterior midline of the fish crossed the threshold of the refugium (Meuthen et al. 2019a). Fish that did not emerge within the 10-min period were assigned a maximum emergence time of 600 s.

### Statistics

Data from the time of spawning to the first spontaneous hatching event, the length of the clutch hatching period, and larval size (TL) at hatch met parametric assumptions for normality, linearity, homoscedasticity, and homogeneity of variance. Hatching was compared between the three developmental treatments (i.e., control, egg cue, and adult cue) via separate one-way analysis of variance (ANOVAs), with days to first hatch and hatching duration specified as dependent variables. Larval size was compared among groups using the *lme4* package in R (Bates et al. 2015) via a linear mixed model, with embryonic treatment (adult, egg, or control) assigned as a fixed factor, and clutch identity specified as a random factor. The difference in proportion of fish in each treatment categorized as developed, underdeveloped, or severely underdeveloped was compared using a Fisher’s exact test, followed by pairwise comparisons among groups using a Benjamini–Hochberg correction for multiple comparisons.

Evidence for behavioral carryover effects associated with embryonic rearing environment was assessed using univariate or multivariate general linear models (GLMs) conducted upon the average behavioral values for each clutch. For the open-field trials, total distance moved, the frequency of entries to the central zone, the duration of time spent in the central zone and the latency to first entry to the central zone were analyzed via a multivariate GLM with embryonic treatment (adult, egg, or control) specified as a fixed factor. Preliminary analysis indicated that total distance moved, the frequency of entries to the central zone and the duration of time spent in the central zone did not satisfy parametric assumptions of normality and so these variables were log-transformed prior to analysis. For the refugium trials, the time to emergence from the refugium was analyzed via a univariate GLM with embryonic treatment specified as a fixed factor. Statistical analyses for larval behavioral assays were conducted in SPSS (version 30).

## Results

### Experiment 1: Ontogeny of embryonic stimulus response

**Fig. 2:**
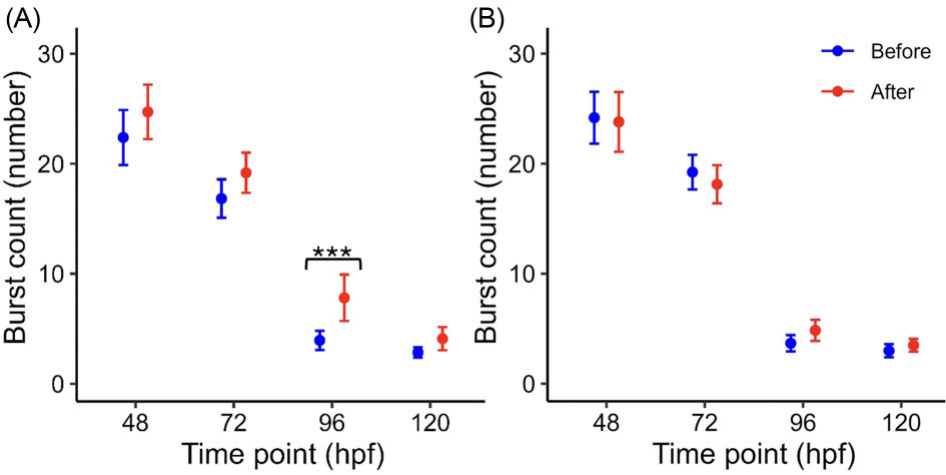
Activity levels (mean ± 95% CI) of embryonic minnows in response to (A) chemical alarm cues from conspecific injured eggs at 48 hpf (*n* = 96), 72 hpf (*n* = 113), 96 hpf (*n* = 89), and 120 hpf (*n* = 72); and (B) chemical alarm cues from adult conspecifics at 48 hpf (*n* = 90), 72 hpf (*n* = 117), 96 hpf (*n* = 80), and 120 hpf (*n* = 81). Significant changes in behavior associated with stimulus presentation are indicated by asterisks: ***P < 0.01.

Across both olfactory cues and time points, a total of 738 embryos were tested for embryonic activity (*n* = 72–117 per group, corresponding to 11 clutches per treatment; see Fig. 2). There was a significant two-way interaction between timepoint and stimulus administration in response to egg alarm cue (Fig. 2A; Table 1), indicating that embryonic responses to external stimuli changed across development. Follow-up comparisons of embryonic responses before and after the administration of stimuli at each developmental timepoint revealed that the embryos in this study showed first evidence of a behavioral response to an environmental olfactory cue at 96 hpf (*Z* = −4.991, *P *< 0.001), by doubling the average activity rate in the presence of egg alarm cue compared to baseline levels (Fig. 2A). In contrast, we did not find evidence of a significant embryonic behavioral response to adult alarm cue at any developmental timepoint (Fig. 2B; Table 1).

**Table 1:** Results of generalized linear mixed models investigating the onset of behavioral responses to olfactory cues from injured conspecific eggs or adults during embryonic development.

	Egg alarm cue			Adult alarm cue		
**Model fixed effects**	*Wald X^2^*	*df*	*P*	*Wald X^2^*	*df*	*P*
Time point	470.05	3	<0.001	662.38	3	<0.001
Stimulus administration	0.86	1	0.35	0.007	1	0.93
Stimulus administration x time point	13.34	3	0.004	6.65	3	0.084

### Experiment 2: Effects of environmental olfactory cues on embryonic development and hatching

**Fig. 3:**
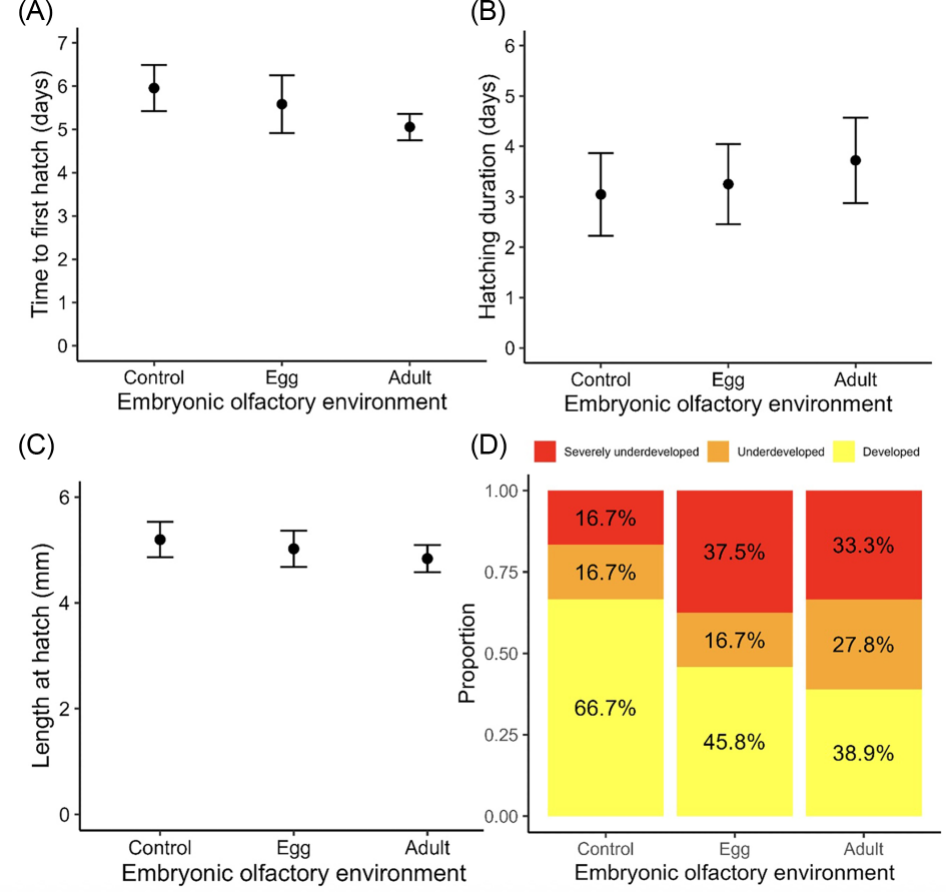
Hatching and development metrics of minnow clutches exposed to chemical cues from injured conspecifics and control eggs. (A) Duration of time (mean ± 95% CI; in days) from the day of spawning to the first evidence of spontaneous hatching. (B) Duration of the clutch hatching period (mean ± 95% CI; in days) from the time of first spontaneous hatch to 90% successfully hatched eggs. Sample sizes are as follows: clutches reared under control conditions, *n* = 11; in the presence of olfactory cues from injured eggs, *n* = 12); or in the presence of adult alarm cue, *n* = 9. (C) The total length of the first 1–3 larvae (mean ± 95% CI; in mm) to hatch from clutches reared under control conditions (*n* = 18), in the presence of olfactory cues from injured eggs (*n* = 24), or adult alarm cues (*n* = 18). (D) The proportions of first-hatched larvae in each treatment (control, egg, and adult cues) classified as severely underdeveloped, underdeveloped, or fully developed based on the nine morphological traits shown in Fig. 1. No significant differences between the groups were found.

A total of 35 clutches were collected and used for this experiment. Due to the timing of some assays, not all clutches were used for all parts of the study. Thirty-two clutches were assessed for the timing of hatching (control: *n* = 11; egg cue: *n* = 12; and adult cue: *n* = 9). Overall, embryos that developed in the presence of olfactory cues indicative of predation risk showed similar patterns of development and hatching to those reared in control conditions. There was no significant effect of embryonic exposure to olfactory cues on the time from spawning until the first spontaneous hatching event (*F*_2, 29 _= 2.29, *P *= 0.12; Fig. 3A). The average time (±SD) until first hatch varied from a minimum of 5.0 ± 0.46 days for clutches exposed to adult alarm cues to 5.95 ± 0.91 days for control clutches. There was also no significant effect of treatment on the length of the clutch-hatching period after the first hatching event was observed (*F*_2, 29 _= 0.62, *P *= 0.54; Fig. 3B).

Twenty-nine clutches were used to assess the effects of the embryonic olfactory environment on the stage of development at hatching (control: *n* = 9; egg cue: *n* = 11; and adult cue: *n* = 9). We did not find a significant effect of embryonic treatment on larval size (TL) at the time of hatching (χ^2^ = 0.20, *df* = 2, *P* = 0.9; Fig. 3C). The average lengths (± SD) of larvae in the control, egg, and adult cue groups were 5.20 ± 0.72 mm, 5.02 ± 0.86 mm, and 4.84 ± 0.56 mm, respectively. Individual development scores of the first hatchlings ranged from 0 to 9, with individuals classified as severely underdeveloped (stages 28–29) characterized by the presence of a large, circular, yolk-sac; a curved longitudinal body axis; the head oriented at an angle less than 140° to the notochord at the point of intersection; a lack of melanocytes, dip in the median fin fold, swim bladder, and yellow bile; and having the dentary bone of the lower jaw still attached to the body. In contrast, fully developed larvae (stages 31–32) had a reduced and elongated yolk sac; a straightened longitudinal body axis; the head oriented to the notochord at an angle larger than or equal to a 140° at the point of intersection; visible melanocytes, swim bladder, and yellow bile; and a detached dentary bone of the lower jaw, capable of opening and closing. Two thirds of the fish examined in the control group were considered fully developed, whereas only 46% of larvae exposed to injured egg cues during embryogenesis, and 39% of larvae exposed to adult alarm cues, were classified as such (Fig. 3D). There was a marginally significant overall effect of developmental environment on the proportions of first-hatched larvae classified as developed, underdeveloped, or severely underdeveloped (Fisher’s test; *P *= 0.05). However, pairwise comparisons conducted among embryonic treatments did not find that these differences reached significance (all *P*s > 0.05).

### Effects of the olfactory environment on behavior after hatching

**Fig. 4:**
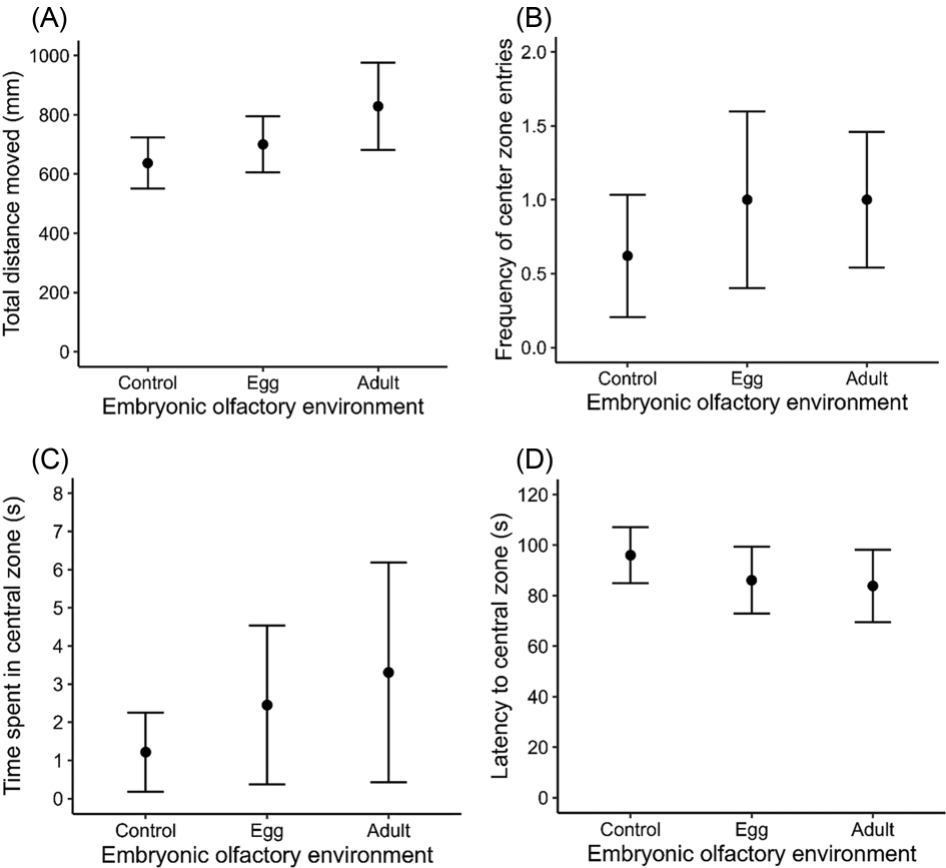
Swimming performance and space use of 21-dpf larvae reared with embryonic exposure to alarm cues from injured eggs (*n* = 50 from 10 clutches), conspecific adults (*n* = 47 from 10 clutches), or in the absense of these cues (control, *n* = 50 from 10 clutches). (A) Total distance moved (mm) in open-field trials (mean ± 95% CI). (B) Frequency of entries into the center zone of the arena (mean ± 95% CI). (C) Total duration of time (s) spent in the center zone of the arena (mean ± 95% CI). (D) Latency (s) to first enter the center zone of the arena (mean ± 95% CI). No significant differences between the groups were found.

A total of 147 larvae were tested in open-field trials (control: *n* = 50; injured egg cue: *n* = 50; and adult alarm cue: *n* = 47) and 146 larvae were tested in refugium emergence trials (control: *n* = 49; injured egg cue: *n* = 50; and adult alarm cue: *n* = 47). Each group contained larvae from 10 clutches. Overall, control larvae and those reared as embryos with exposure to different conspecific alarm cues showed similar behavior after hatching (Pillai’s Trace: 0.38, *F*_8,50_ = 1.49, *P *= 0.19). In open-field trials, the mean (±SD) total distance moved varied from a minimum of 637.02 ± 201.76 mm for larvae in the control group to a maximum of 843.22 ± 465.69 mm for those exposed to adult alarm cues as embryos (Fig. 4A). However, these differences among the groups in swimming distance were not statistically significant (*F*_2,27_ = 1.08, *P *= 0.36). Although larvae from the two alarm cue treatment groups spent at least twice as much time on average in the center zone than those in the control group, larvae from the three developmental treatments showed statistically similar patterns of space use; focal fish in all groups entered the center zone with similar frequency (*F*_2,27_ = 0.99, *P *= 0.38; Fig. 4B), and spent a similar minority of trial time in the central zone of the arena (*F*_2,27_ = 2.96, *P *= 0.07; Fig. 4C). There was also no effect of embryonic treatment on the latency of larvae to first enter the center zone (*F*_2,27_ = 0.62, *P *= 0.55; Fig. 4D).

**Fig. 5:**
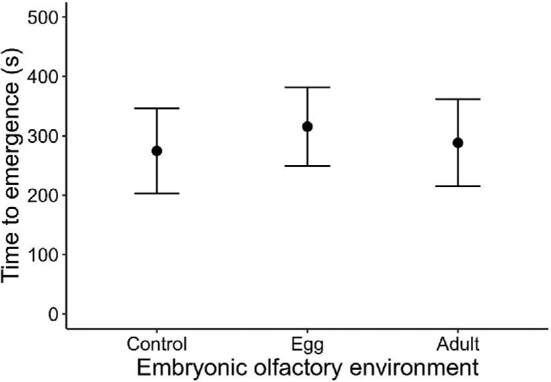
Mean ± 95% CI latency (s) to emergence from a refugium by 21-dpf larvae reared with embryonic exposure to chemical cues from injured eggs (*n* = 50 from 10 clutches) or conspecific adults (*n* = 47 from 10 clutches), or that developed in the absence of these cues (control, *n* = 49 from 10 clutches). No significant differences between the groups were found.

In the refugium emergence trials, 48 of 146 total tested individuals did not emerge from the refugium by the end of the 10-min trial period (control: 16/49; egg: 16/50; and adult: 16/47). Overall, the clutch-averaged time to emergence was statistically similar among groups (*F*_2,27_ = 0.16, *P *= 0.85), ranging from a minimum average (±SD) of 280.36 ± 171.53 s for larvae in the control group to 315.5 ± 173.18 s for those exposed to cues from injured eggs during embryogenesis (Fig. 5).

## Discussion

The influence of the prenatal sensory environment on behavioral development is an area of growing scientific interest (Lickliter 2018; Du and Shine 2022). In this study, we conducted a time-series experiment using embryonic fathead minnows from 48 to 120 hpf (approximately stages 25–32; Devlin et al. 1996) to identify the developmental window(s) during which embryos begin to exhibit behavioral responses to environmental cues that contain different information about predation risk (i.e., predation upon free-swimming conspecifics vs. injured eggs); and to evaluate whether clutches exposed to these cues before hatching behave differently after hatching compared to those raised in the absence of cues. Our results indicate that embryos begin to respond to environmental olfactory cues indicative of predation by 96 hpf. Notably, embryos exhibited observable changes in activity in response to chemical cues derived from injured conspecific eggs at 96 hpf but not to those from adults. However, pre-hatch exposure to conspecific alarm cues did not result in statistically significant plasticity in development, timing of hatching, or post-hatch behavior. These findings indicate that although fathead minnow embryos can detect and respond to environmental information relevant to predation, pre-hatch exposure to chemical alarm cues alone may not necessarily influence hatching or post-hatch patterns of behavior.

In this study, an increase in embryonic activity in response to chemical cues from crushed eggs was observed at 96 hpf, indicating the onset of olfactory-mediated behavioral responses by this time. Such responses to conspecific alarm cues are well documented across a variety of aquatic taxa, including fishes, amphibians, and members of the Mollusca, where they function as an important elicitor of antipredator behavior (Ferrari et al. 2005; Mirza et al. 2006; Wasserman et al. 2014). In fishes, olfactory processing begins with the detection of odorant molecules by receptors in the epithelium of the olfactory pits. These signals are then transmitted via the olfactory bulb to higher-order brain regions (Hara 1975). In fathead minnows, the olfactory pits begin to form at approximately 26 hpf, neural connections between the olfactory bulb and cerebrum are established by 74 hpf, and additional sensory cells appear in the olfactory groove by 85 hpf (Devlin et al. 1996). It is possible that olfactory *perception* may occur earlier in development, given that this study was concerned with identifying when *responses* to olfactory stimuli are first observed. However, given the developmental timeline of this species we suggest that the observed absence of significant olfactory responses at 72 hpf or earlier reflects incomplete maturation of the sensory and neural circuitry required for odor detection and processing rather than an inability to produce a response.

Although embryos responded to chemical cues from conspecific eggs, they did not exhibit significant behavioral changes in response to a similar olfactory cue derived from conspecific adults. This finding was initially unexpected, because embryonic behavioral responses to such cues have been reported both before and after hatching; for example, Crowder and Ward (2022) reared embryos in the continual presence of adult alarm cues and found that individuals exhibited reduced activity compared to control embryos before hatching at 5 dpf and a lower level of baseline swimming activity after hatching. However, at least some of this discrepancy may be attributed to differences between the two studies in methodology; specifically, the embryos in this study were reared under control conditions and not in the continual presence of any predator-related cues. One possibility for the observed difference in independent patterns of embryonic responses to adult and egg cues that we observed could be that these cues provided different informational content to the individuals exposed to them. Specifically, chemical indicators of egg predation may be interpreted as a more immediate or relevant threat to the individuals at the egg stage, especially because individuals at different developmental stages may occupy different prey guilds and face different predation pressures (Horn et al. 2019; Horn and Chivers 2021a). Because differences in production techniques make it difficult to quantify the precise concentration of alarm cue generated from fishes for comparative purposes, additional research is necessary to unequivocally test the hypothesis that embryos are more sensitive to olfactory cues from eggs than adults. However, this interpretation is consistent with the general hypothesis that prey should modify their responses based on the perceived level of risk associated with a given cue (Chivers et al. 2002; Lönnstedt and McCormick 2011; Mitchell and McCormick 2013).

We also investigated whether fathead minnows exhibit plasticity in embryonic development and the timing of hatching associated with repeated exposure to chemical cues from injured conspecific adults or eggs. Previous research on fishes (Latham and Just 1989; Kusch and Chivers 2004; Wisenden et al. 2022), amphibians (Warkentin 1995; Mandrillon and Saglio 2009; Jung et al. 2020), and invertebrates (Li 2002) provide compelling support for the hypothesis that embryos modulate the timing of hatching in response to perceived environmental threats. However, the direction of this plasticity (i.e., whether hatching is accelerated or delayed) is often dependent on the nature and intensity of the threat (Ireland et al. 2007; Warkentin 2011b; Güell et al. 2022). For example, (Ireland et al. 2007) found that green frog (*Rana clamitans*) embryos hatched earlier when exposed to egg predator cues but delayed hatching in response to cues from larval predators. Cue-specific hatching plasticity has also been reported in fathead minnows, with embryos exposed to egg-predation cues hatching earlier and at a smaller size than controls (Kusch and Chivers 2004), whereas those exposed to cues from injured adults did not (Horn and Chivers 2021b; Crowder and Ward 2022). In this study, embryos exposed to both egg and adult olfactory cues tended to hatch earlier than controls, and the first embryos to emerge from the alarm cue treatments were less-developed, but the trends did not reach statistical significance. It is possible that early-hatching individuals differ morphologically from later-hatching siblings, and future studies could include additional samples from across the full hatching period to better characterize this plasticity. However, one explanation is that in minnows, as in other species (Ferrari et al. 2010b; Warkentin 2011a; Du and Shine 2022), the potential benefits of early hatching in response to predators may be offset by increased mortality after hatching by less-developed individuals. In such cases, embryos may require the integration of multiple reliable environmental cues that signal predation risk (i.e., chemical cues and mechanical cues) before initiating hatching (Wisenden et al. 2022).

Exposure to conspecific alarm cues during the egg stage also did not significantly influence larval behavior in either open-field or refugium-emergence assays. Previous research examining behavioral carryover effects of embryonic experience have yielded mixed results, some of which align with our findings. For example, Crowder and Ward (2022) reported that larvae exposed to adult conspecific alarm cues during embryogenesis exhibited increased freezing behavior compared to controls, which is widely accepted to represent an antipredator response (Fuiman and Magurran 1994). Similarly, Mathis et al. (2008) found that embryos reared in the presence of older, predatory conspecifics were less active after hatching. However, other studies have reported no evidence of behavioral carryover effects following embryonic exposure to conspecific alarm cues alone (Horn and Chivers 2021a; Nelson et al. 2013). In our study, one possible explanation for the absence of observable differences in behavior in larvae exposed to cues from injured eggs is that this cue was perceived as irrelevant after hatching—a phenomenon known as “adaptive forgetting” (Ferrari et al. 2010a). Such forgetting may be advantageous if it prevents unnecessary allocation of energy and neural resources toward irrelevant threats, allowing individuals to prioritize the acquisition of new and more developmentally appropriate information (Ferrari et al. 2019; Horn et al. 2019). Alternatively, post-hatching behavioral plasticity associated with the embryonic sensory environment may only be expressed in the presence of those cues.

The fathead minnow is an emerging model organism for investigations of embryonic learning and memory, sensory ecology, communication, phenotypic variation, and behavioral development (Kusch and Chivers 2004; Böhler 2021; Horn and Chivers 2021a, 2021b; Crowder and Ward 2022; Karasch and Ward 2025). Our results add to this growing body of literature by identifying the developmental windows during which embryos begin to respond to olfactory environmental stimuli. Additional behavioral and/or neuroimaging studies are now needed to (1) more precisely determine the specific stage(s) of development at which these responses first emerge and (2) disentangle the initial onset of embryonic sensory perception from that of behavioral response. In addition, our results provide novel evidence indicating that fathead minnow embryos can discriminate between olfactory cues that differ in informational content and that prior to hatching they are behaviorally sensitive to cues that indicate predation upon eggs. Taken together, these results suggest that aquatic embryos are capable of more sophisticated environmental assessment abilities and decision-making than has previously been assumed.

## Data Availability

The data are available in *Dryad* at DOI: https://doi.org/10.5061/dryad.kh18932m4.
